# Editorial: The Role of Biomembranes and Biophysics in Immune Cell Signaling

**DOI:** 10.3389/fimmu.2021.740373

**Published:** 2021-09-24

**Authors:** Yan Shi, Erdinc Sezgin, Wei Chen

**Affiliations:** ^1^ Department of Basic Medical Sciences, Institute for Immunology, Beijing Key Lab for Immunological Research on Chronic Diseases, School of Medicine, Tsinghua University, Beijing, China; ^2^ Department of Microbiology, Immunology and Infectious Diseases, Snyder Institute, University of Calgary, Calgary, AB, Canada; ^3^ Science for Life Laboratory, Department of Women’s and Children’s Health, Karolinska Institutet, Solna, Sweden; ^4^ Department of Cell Biology and Department of Cardiology of the Second Affiliated Hospital, Zhejiang University School of Medicine, Hangzhou, China

**Keywords:** cell membrane, lipid domains, membrane biophysics, receptor signaling, T cell receptor

The plasma membrane is the ultimate demarcation between “in” and “out” for cellular life forms. It is also the platform on which “cross border” communications take place, such as receptor ligand interactions. Here, several thousand species of membrane proteins anchored by their transmembrane domains on this platform specifically sense complex biological, biochemical, and biophysical cues in the extracellular environment and trigger signaling cascades to determine various cellular functions. While modern technologies permit our study into the structure, dynamic, and function of these proteins, the reality is, the behaviors of this platform itself are likely more complex and in most cases heavily shielded from our probing eyes. The challenge of studying how membrane properties affect membrane-receptor functions is multi-faceted, and this is rooted in the complex heterogeneity of the membrane itself. As each layer of complexity can affect cellular signaling, the question becomes where we shall start to look. One of the issues is the limited availability of suitable tools. The most evident one is the resolution power constrained by optical diffraction, the size below which most biophysical events take place on the membrane. Another challenge is that the tools required for biophysical sensing and manipulation are often of limited sensitivity of mechanical forces. In addition, different from protein and nucleic acid related research, whereby clear causality can be established when all variables are controlled, membrane biophysics is intrinsically chaotic with numerous factors working at the same time in a promiscuous manner and events are often transitional.

In this Research Topic collection, the coverage reflects the complexity of this battle ground. Significant attention is paid to the instrument development that is arguably more important to this research scheme than conventional biochemistry-based research fields. In this editorial, we attempt to establish a progressive line based on three dimensions of complexity in biomembrane research, and settle the contributors’ insights into suitable spots on this logic chain.

Singer and Nicolson’s mosaic model is straight forward: that lipid species are randomly distributed and proteins are present free from additional constraints. This view, however, is too simplistic. It is generally accepted that membrane and membrane proteins have several layers of complexity. 1. Heterogeneity of membrane bilayer. 2. Lipid-protein interface and secondary membrane structure. 3. Receptor signaling in a lipid environment.

First layer of complexity comes from the lipid domains. One of the key features of eukaryotic cell forms is the emergence of cholesterol synthesis, typically *via* mevalonate pathway in animals. This simple lipid species changes the membrane behavior substantially and is a key player on the bilayer asymmetry. In mammalian membrane, due to their peculiar packing requirement, sphingolipids are only present on the outer leaflets. The presence of sphingolipids and cholesterol forms the first layer of complexity: lipid ordered domains, roughly equivalent of lipid rafts. Physiological ordered domains on a cell surface likely involve coupling between outer and inner leaflets, cytosolic components such as cytoskeleton and several classes of transmembrane and membrane-associated proteins. However, the fact remains that in giant unilamellar vesicles with defined ternary mixtures of saturated and unsaturated lipids with cholesterol, the phase separation still takes place. Intriguingly, on >1000 component giant plasma membrane vesicles (cell-derived vesicles that preserve largely cellular plasma membrane composition), phase separation is still observed and the ordered domains to some degree can be regarded as “raft”-like (although larger). The domain behavior in model systems (such as size, shape etc.) can be modulated *via* changing the environmental conditions or adding external components (1–3). The ordered domains have several essential features: they probably sort the inner leaflet lipid species across the hydrophobic core, yet this interleaflet coupling still needs extensive work both in model systems and in live cell membranes. In this regard, role of membrane asymmetry on membrane domains is also largely unexplored. Recent work shows that plasma membrane is asymmetric not only in lipid head groups but also in acyl chain length and saturation (4). Therefore, it will be exciting to see future work on how this multi-layer asymmetry affects the interleaflet coupling, domain formation and their physicochemical properties. The ordered domains are also assumed to be linked to cortical cytoskeleton *via* linker such as ERM (ezrin/radixin/moesin) as a result of inner leaflet lipid tuning. In laying the biophysical grounds to establish a model that explains microvilli on T cells, Cebecauer provided a sophisticated overview how this may work. Sphingolipid-dependent lipid domains are distinguished from the rest of the membrane by a lipid-lipid interface holing a definitive line tension. This tension creates a circular entrapping effect that drives lipid domains upwards. The accumulation of PIP2 likely draws in FERM (Band 4.1 ERM)-domain containing proteins that drive the growth of microvilli on T cells. The underlying principle is likely applicable to ordered domains without invoking the extreme curvature as in microvilli formation. Taking the angle of membrane cholesterol, Zhang et al. discussed how receptors may be regulated by this lipid domain. A key point, even from the narrow confines of cholesterol, is that its impact on receptor signaling can be extremely complex and stimulation-dependent. Cholesterol by itself inhibits TCR β chain from entering a “primed” state. On the other hand, it is also essential to potentiate T cell functions probably through enhancing TCR and lipid domain clustering. Such regulation is likely involved in BCR and FCR signaling. From the biochemical standpoint, cholesterol stiffens the local lipid environment, which may be a key platform for the receptor ligation to transduce mechanical force across the membrane. From a theoretical perspective, Lamerton et al. discussed the concept of protein and lipid clustering. At the lipid level, clustering may be lipid rafts from a different viewing angle, particularly when defined as saturated acyl tails working together with cholesterol to form densely packed lipid domains.

The second layer of complexity is the interaction of lipid membrane and proteins. Proteins in this case can be separated into two groups per their association with the membrane. The first group is those contained within the bilayer, such as Bin-Amphiphysin-Rvs family proteins that intrinsically alter membrane curvature. They interact with cytoskeletal regulators which control actin nucleation and F-actin remodeling. Realistically, all transmembrane proteins likely impact biophysical properties of lipid membranes to various extents, however, we shall refrain from that discussion as the reciprocity therein becomes too compounded for this editorial. The second group of proteins are those approaching the membrane from the inside, mainly those of cortical cytoskeleton. Chief among which are the ERM proteins. Their central involvement can be deduced from their structure. N-terminal FERM domains are known to bind a diverse set of cytosolic tails of transmembrane proteins, such as CD44 and EGFR. Critically, FERM domain has specificity to PIP2 (phosphatidylinositol (4,5) biphosphate), a lipid species known to sort per influence of lipid orders. The C-terminal of ERM proteins is a C-ERMAD domain, which binds to F-actin. FERM and C-ERMAD domains self-associate and remain soluble in cytosol. Binding of FERM to PIP2 activates those proteins. This design creates numerous interactions as a consequence of membrane dynamics on the inner leaflet and is likely responsible for the more complex membrane protrusions, such as filapodia, lamina podia, and as the “favorite child” of this collection, T cell microvilli. Orbach and Su presented an abstract, yet vivid, reconstitution on how those microvilli come to being on T cells surface. The role of those often ignored features in T cell biology is also convincingly illustrated and it is understood that such a finger-like feature may increase the “scanning” of antigen presenting cells. From a different perspective, these membrane protrusions may represent an underappreciated hand in organizing lipid domains and receptor complex components, which may have different preferences to a particular lipid order. A key event described by the kinetic-segregation model is the separation of CD45 from the rest of signaling complex, which likely maintains a state of high phosphorylation for the key factors associated with TCR/CD3 signaling. Apparently, structural features of microvilli favor this segregation. Of particular interest is the piece by Dam et al. that discussed a phenomenon related to this topic (5). Supported lipid bilayers are commonly used a surrogate of MHC/peptide to interact with TCR. Such a design had led to an unexpected finding that exclusion of CD45 from the TCR complex is sufficient to trigger ligand free activation. Dam et al.’s data suggested that this might be due to intrinsic binding to nickel-chelating lipids, rather than specific TCR interactions, which serves a reminder that some biophysical analyses must be carefully controlled to avoid artifacts.

The third layer of complexity is how lipid environment impacts receptor signaling. Starting from a resting receptor, there are two parameters to be determined. 1. Their preference with reference to ordered domains. 2. Their state of aggregation prior to ligand binding. Upon ligand binding, biophysical properties of the membrane will significantly alter receptors’ signaling potential. In the case of TCR, the story is complex. For a simple treatment of cholesterol depletion, T cell activation can be either diminished or enhanced. This outcome is setup-dependent, revealing that there is much more information yet to be extracted. Likely, the association of TCR β chain with cholesterol is inhibitory (this is still being debated). The activation step requires a step that escapes this association. Yet, src family kinases are located in ordered domains, and at minimum final assembly of signaling complex requires proper arrangements of TCR, CD3 chains, transmembrane phosphatases, upstream ITAM-specific kinases, downstream targets including LAT platform, as well as several enzymes involved in PI metabolism. If the lipid order is integrated into TCR signaling, there must be some type of rearrangement of each component, not only for their positioning against each other, but also for their interfaces toward the lipid environment – a complexity likely beyond our current comprehension. Equally important, the formation of the signaling complex is also regulated by the cytoskeleton, which to some degree controls the formation of transmembrane receptors. Either through individual dissection, or a grand design of a formidably intricate experiment, solving the details of this process will be the final answer to TCR activation. At the moment, we know the lipid composition is critical to TCR activation. For instance *the in-situ* TCR-pMHC binding strength is likely lipid-dependent (Zhang et al.). This step perhaps introduces a conformational change that turns both TCR ectodomains and the CD3 chains, exposing ITAM motifs folded into negatively charged inner leaflet. Membrane bilayer could also provide physical platform to produce and transmit biomechanical force to regulate TCR antigen recognition by inducing TCR/CD3 and pMHC conformational changes and to trigger signaling. As to be expected, biophysical properties can affect receptor signaling, thus impacting several categories of immune dysfunctions and diseases. In this series, Gunasinghe et al. gave a comprehensive review that can serve as an insightful reference for those interested in biomechanical dysfunctions in chronic diseases. Shi and Ruan presented another possibility in addition of organizing role of ordered domains in receptor signaling, that the presence of lipid domains may be an intrinsic property that prevents receptor from spontaneous activation. Receptor activation may in some cases a simple instruction from ligands to avoid this suppression. Whether this proposal can stand the test of time will depend on experiments of several major receptor ligand interactions, especially with the aid of recent instrument development.

With all the challenges discussed, we remain enthusiastic that biophysical research on membrane is entering a new era. This confidence is supported by the rapid arrival of new instruments. Compared with other research fields, instrument and protocol developments are the lifeline of new discoveries. A sobering example is the transition in membrane domain research from the sole dependence of detergent solubilizing extraction to polarity sensing lipid dyes such as laurdan, as well as, sub-optical diffraction imaging, as illustrated by Lamerton et al. in this series. With a focus on cytoskeletal interaction with plasma membrane, Schneider et al. discussed at depth the new imaging tools currently available, starting from more conventional TIRF, SPT to more sophisticated fluorescence fluctuation-based FCS in combination with STED and fluorescence energy transfer-based techniques. This discussion should be of considerable value in helping select best and latest tools for all of us. Another interesting piece by Stanly et al. discussed a new development in optical diffraction tomography in studying intracellular sphericity, as well as refractive index in diseased platelets, pointing to an uncommon biophysical angle of evaluation (6). Using FLIM and FRET techniques, Vleeshouwers et al. presented new findings on how prostaglandin E2 receptors regulate podosome dissolution *via* microtubules. While this study is not directly focused on biomembrane, it nevertheless reflects the additional dimension biophysics in cell biology, such as how both two systems of cytoskeleton, actin and microtubules, take part in cellular adhesion.

In the original proposal to start this Research Topic, it was our intension to gather thoughts on biophysical analyses of cellular membranes, as the area is relatively uncoordinated in protocols, standards and concepts. It was recognized that coordination can only be started with a substantive exchange. At this moment of closing off this series, we are excited by the breadth and insight of submissions collected. This effort, in our view, is one of the very rare series solely focused on biophysics on cellular membrane and a good foundation for future growth of this research scheme.

## Author Contributions

All authors contributed equally. All authors contributed to the article and approved the submitted version.

## Conflict of Interest

The authors declare that the research was conducted in the absence of any commercial or financial relationships that could be construed as a potential conflict of interest.

## Publisher’s Note

All claims expressed in this article are solely those of the authors and do not necessarily represent those of their affiliated organizations, or those of the publisher, the editors and the reviewers. Any product that may be evaluated in this article, or claim that may be made by its manufacturer, is not guaranteed or endorsed by the publisher.
